# Impact of egg quality on hatchability of poultry hatching eggs: research progress and regulatory strategies

**DOI:** 10.1016/j.psj.2026.107314

**Published:** 2026-06-17

**Authors:** Yihan Wang, Zeyao Zhang, Xuefeng Shi

**Affiliations:** College of Animal Science and Technology, GuangXi University, Nanning 530004, China

**Keywords:** Egg quality, Hatchability, External quality, Internal quality, Storage management

## Abstract

Egg hatchability is a key indicator of reproductive efficiency in poultry. Egg quality, which serves as the material basis for embryonic development, plays a pivotal role during incubation. Egg quality includes both external and internal components that influence embryonic growth and hatchability through the modulation of gas exchange, water balance, microbial barriers, and nutrient supply. External qualities, including eggshell thickness, strength, and pore structure, determine protective properties and permeability, whereas eggshell color and translucency reflect the structural integrity and mineralization status. Internal quality, including the yolk-to-albumen ratio, albumen freshness (Haugh unit, HU), and albumen physicochemical properties, affects the nutrient supply and stability of the internal environment of the egg. The effects of egg quality components are further modulated by management factors such as storage duration, storage temperature, and incubation conditions. This review summarizes the mechanisms linking egg quality to hatchability and outlines strategies for improving egg quality and hatchability.

## Introduction

The poultry industry is a vital component of the modern agricultural production system; its sustainable development majorly depends on the effective propagation of superior genetic resources (Zhang et al., 2025). The hatchability of eggs is a key indicator used to evaluate the reproductive and economic efficiency of poultry breeding systems (Ori, 2011). Improved hatchability can significantly increase the number of healthy hatched chicks and reduce unit production costs. Moreover, it is crucial for conserving elite genetic resources and the long-term stability of population production performance. However, in commercial production systems, hatchability is influenced by multiple factors, including nutritional and health statuses, feeding and management practices, egg storage conditions, and incubation parameters (Wang, 2021; Adame and Ameha, 2023). Among these factors, hatching egg quality plays a fundamental role in determining embryo survival and normal embryonic development, and is directly associated with hatchability (Xu et al., 2017; Nasri et al., 2020).

Hatching eggs not only serve as carriers of genetic information but also represent the sole microenvironment supporting early embryonic survival and development (Moran, 2007). Consequently, egg quality serves as a biological foundation for successful incubation; moreover, it is generally considered a multidimensional trait that includes external egg quality (eggshell characteristics) and internal egg quality (albumen and yolk characteristics) (Biesek, 2023; Liu et al., 2023). These quality traits collectively regulate the gas exchange, moisture balance, microbial barrier function, and nutrient availability during embryonic development. Previous studies have demonstrated the significant impact of the eggshell thickness, eggshell strength, and eggshell pore structure, as well as variations in internal egg quality, on the embryonic development and final hatching outcomes (Abd El-Hack et al., 2022; Liu et al., 2023). Therefore, systematically elucidating the relationships between different egg quality indicators and hatchability is essential to optimize hatching egg management strategies and improve incubation efficiency (Biesek, 2023).

Although the importance of egg quality in regulating hatchability has been widely recognized, the extent to which different egg quality indicators influence hatching outcomes and the underlying mechanisms remains unclear (Biesek, 2023). Variations in poultry species, breeds, and maternal backgrounds may contribute to the inconsistencies in research findings (Abd El-Hack et al., 2022; Yang et al., 2025). However, egg quality is not a static trait. Its effects are often confounded by several management factors, including storage duration and conditions, and incubation conditions, thereby increasing the complexity of interpreting research findings and applying them in production (Biesek, 2023; Yalcin et al., 2022). Therefore, the integration of existing research evidence from a comprehensive perspective is necessary to evaluate the effects of different egg quality components on hatchability, clarify the underlying mechanisms, and improve the interpretation and application of current findings under diverse production conditions (Yang et al., 2025).

Although previous reviews have addressed specific egg quality traits or incubation-related factors(Abd El-Hack et al., 2022; Biesek, 2023), an integrated understanding of how external and internal egg quality characteristics interact with environmental factors to influence hatchability remains limited. Therefore, this review comprehensively summarizes current evidence on the relationships between hatching egg quality and hatchability in poultry, aiming to clarify the underlying mechanisms and reconcile inconsistent findings across studies. Particular attention is given to the interactions among egg quality traits and management factors that collectively influence embryonic development and hatching success.

**Fig. 1:**
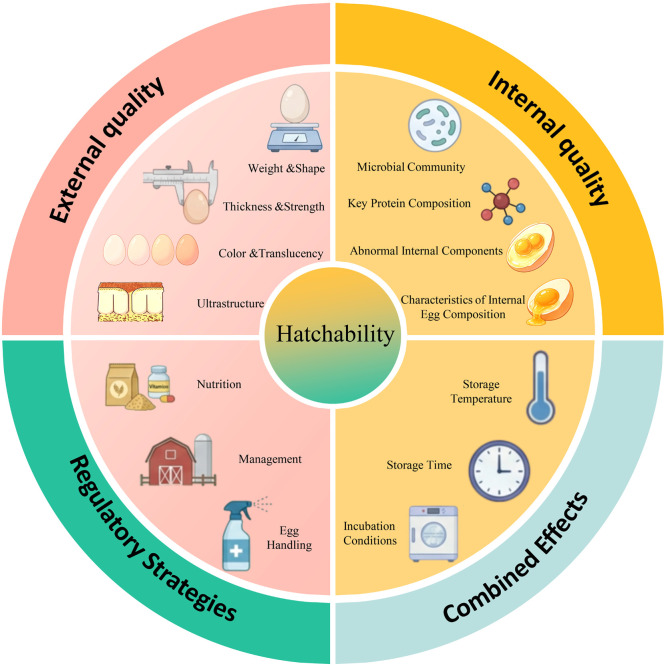
Overview of the relationships between egg quality and hatchability in poultry. External egg quality and internal egg quality directly affect embryonic development, while storage conditions and incubation parameters further modulate these effects. Management and nutritional strategies can improve egg quality and optimize incubation outcomes, thereby enhancing hatchability.

To facilitate a systematic understanding of these relationships, an integrated framework is presented in Fig. 1. As illustrated, hatchability is jointly regulated by external egg quality (e.g., eggshell characteristics), internal egg quality (e.g., egg composition and microbial community), and environmental and management factors such as storage conditions and incubation parameters. These factors interact to influence key biological processes, including gas exchange, water balance, microbial barrier function, and nutrient supply, ultimately determining embryonic development and hatching success.

## Evaluation indicators of egg quality

Egg quality encompasses both external and internal attributes (Moreki et al., 2025; Yang et al., 2025). External egg quality mainly includes egg weight, egg shape index, eggshell color, eggshell thickness, eggshell strength, specific gravity, eggshell pore characteristics, and shell surface cleanliness (Moreki et al., 2025; Yang et al., 2025). Previous studies have further emphasized that eggshell thickness, shell breaking strength, and egg shape index are among the most widely used indicators for evaluating external egg quality, whereas albumen height, Haugh unit, and yolk color are considered representative measures of internal egg quality and egg freshness(Roberts, 2004; Küçükyılmaz et al., 2012; Rho et al., 2024).The eggshell acts as a mechanical barrier that protects the embryo and performs critical functions in moisture retention and gas exchange; therefore, the structure and integrity of the eggshell directly influence the embryonic survival rate (Biesek, 2023; Gautron et al., 2021). In contrast, internal egg quality reflects the physicochemical properties and freshness of the egg contents, and primarily includes albumen height, Haugh unit, yolk proportion, and the nutritional and chemical composition of the yolk (Nhan, 2025; Rodríguez-Mengod et al., 2024). In addition, the egg microbiota and genetic variations in key egg proteins are closely associated with embryonic development and hatching outcomes (Jin et al., 2022; Wang et al., 2019). Emerging evidence indicates that eggshell microbiota may serve as an additional biological indicator of incubation outcomes, as appropriate microbial control can reduce shell contamination without adversely affecting hatchability (Taşdemir et al., 2021; Coulibaly et al., 2023).

## Effects of external quality on hatchability

The external egg quality influences the mechanical protective properties and gas exchange capacity of eggshells, thereby providing essential physical protection during embryonic development. As illustrated in Fig. 1, external egg quality, particularly eggshell characteristics, plays a fundamental role in regulating key processes such as gas exchange, water balance, and microbial barrier function, which are critical for maintaining a stable environment for the developing embryo. Previously, the relationship between external egg quality and hatchability was investigated from multiple perspectives, including the macroscopic morphological characteristics of the egg, eggshell mechanical properties, and eggshell microstructure.

### Egg weight and egg shape index

Egg weight is one of the most commonly used indicators of external egg quality during the grading and selection of hatching eggs and is closely associated with embryonic developmental space and the proportional composition of egg content (Abebe et al., 2023; Yao et al., 2022). However, the relationship between egg weight and hatchability remains inconsistent across studies. Some reports have suggested that increased egg volume may reduce the efficiency of gas exchange and heat transfer during incubation, thereby adversely affecting normal embryonic development (Cavero et al., 2011). In contrast, other reports have indicated that increased egg weight potentially improves hatchability and suggested that a larger eggshell surface area and greater nutrient availability may enhance embryonic development (Kostaman and Sopiyana, 2021). These discrepancies may be attributed to differences in poultry species and breeds, breeder age, egg size distribution, and incubation management conditions (Yamak et al., 2023). For example, heavier eggs produced by young breeder flocks often contain greater nutrient reserves without substantial deterioration in shell quality, whereas excessively large eggs from older breeders may exhibit thinner shells, impaired gas conductance, and increased embryonic mortality (Cheng et al., 2023; Junior and Pereira, 2024). Accumulating evidence suggests that excessively large eggs have relatively thin shells, reduced yolk absorption efficiency, and increased late embryonic mortality, all of which collectively contribute to reduced hatchability. In contrast, moderate weight of hatching eggs is generally associated with better structural integrity of the eggshell and coordinated embryonic development, which results in relatively higher hatchability. Previous studies have reported that the optimal egg weight range varies among poultry species and genetic lines. For instance, hatchability in broiler breeder eggs is generally higher in eggs of intermediate weight (approximately 52–68 g), whereas excessively small or large eggs tend to exhibit reduced hatchability (Wilson, 1991). Similarly, studies in laying hens and local chicken breeds have suggested that medium-sized eggs generally achieve superior hatching performance compared with excessively small or large eggs (Nowaczewski et al., 2022). This finding validates that egg weight has a breed- and production-condition-specific optimal range, which reflects that both excessively large and excessively small eggs are unfavorable for successful hatching (Ipek and Sozcu, 2017; Nowaczewski et al., 2022). In addition, flocks with greater uniformity in egg weight generally exhibit higher overall hatchability (Bouba et al., 2021).

The effect of the egg shape index, typically defined as (egg width/egg length) × 100, on hatchability is less than that of egg weight. Most studies indicate that the egg shape index, per se, does not exhibit a significant direct association with hatchability (Kostaman and Sopiyana, 2021). Nevertheless, some reports demonstrated a positive correlation of the egg width with hatchability, whereas the egg length and overall egg shape index did not exhibit significant effects (Abanikannda et al., 2025). In commercial poultry production systems, normally shaped hatching eggs constitute the majority; this population-level morphological uniformity may facilitate stable hatchability. Therefore, egg shape index is generally considered a supplementary morphological indicator, and its influence on hatchability may be expressed through interactions with other egg quality traits.

### Eggshell thickness and eggshell strength

The eggshell serves as a physical barrier for embryonic development, and its thickness and mechanical strength play important roles in maintaining normal embryonic development (Narushin and Romanov, 2002; Rayan and Fathi, 2026; Rayan et al., 2022). A thicker and stronger eggshell facilitates stable mechanical protection, reducing the risk of breakage and microbial invasion during handling and incubation. It can also reduce excessive water loss during incubation owing to the longer pore pathway; hence, it exhibits a significant positive association with hatchability (Liao et al., 2013). Moreover, the longer diffusion pathway provided by a moderately thicker shell may hinder microbial penetration and help maintain a more stable microenvironment for embryonic development. However, the beneficial effects of eggshell thickness are not unlimited. Excessive shell thickness may reduce eggshell conductance by restricting the diffusion of oxygen and carbon dioxide through shell pores, thereby impairing embryonic respiration and metabolism, particularly during the late stages of incubation when oxygen demand increases rapidly. (Berrang et al., 1999; Rahn et al., 1987)

Previous studies have suggested that eggshell thickness within an appropriate range is generally associated with improved hatchability (Cheng et al., 2023; Yamak et al., 2023). In commercial chicken breeding systems, eggshell thickness generally ranges from approximately 0.30 to 0.40 mm. Within this range, the shell is considered capable of providing sufficient mechanical protection while maintaining adequate gas exchange and water loss necessary for normal embryonic development and successful hatchability(Nys et al., 2004; Rahn et al., 1987; Sun et al., 2012). In contrast, an excessively thin eggshell increases the risk of embryonic dehydration and microbial penetration, thereby compromising the survival rate of the embryo (Lunam and Ruiz, 2000). Conversely, eggs with shells thinner than approximately 0.30 mm are more susceptible to breakage, excessive moisture loss, and embryonic mortality, ultimately resulting in reduced hatchability. Some reports have demonstrated a positive correlation between eggshell strength and hatchability of hatching eggs (Cavero et al., 2011). However, the dynamic stiffness of eggshells exhibits a weak negative correlation with hatchability, which may be attributed to the high stiffness-associated low porosity and gas exchange efficiency of eggshell, which affect normal embryonic development (Cavero et al., 2011).

Although egg specific gravity is a commonly used indirect indicator of eggshell thickness and shell density, its influence on hatchability remains controversial. Genetic analyses revealed that egg specific gravity is positively associated with hatchability, corroborating that a denser eggshell structure is beneficial for embryonic development (Rozempolska-Rucińska et al., 2011). However, other studies reported no significant relationship between egg specific gravity and hatchability, which may be attributable to differences in sample sources, breed characteristics, and measurement methods (Peebles and Brake, 1987). Macroscopic indicators, such as eggshell thickness, eggshell strength, and egg specific gravity, can, to a certain extent, influence the mechanical protection and physiological support of the embryo, thereby effectively reducing the egg breakage rate and risk of pathogenic microbial invasion during egg storage and incubation (Peebles and Brake, 1987; Rozempolska-Rucińska et al., 2011). However, a single indicator is insufficient to fully predict hatching outcomes, as its effects may be modulated by the eggshell pore structure and microstructure.

### Eggshell color and translucency

Eggshell color is an important phenotypic characteristic of external egg quality in poultry, which is primarily regulated by the deposition patterns of pigments, such as biliverdin and protoporphyrin, in the shell gland during eggshell formation (Lu et al., 2021). Eggshell color and pigment distribution significantly differ across species and breeds. Previous studies have suggested that eggshell color cannot directly determine hatchability; however, under certain conditions, variations in shell color, which reflect eggshell structural uniformity, degree of shell mineralization, or maternal physiological status, may indirectly influence hatching performance (Gu et al., 2025; Ismael et al., 2024; Neto et al., 2024; Nowaczewski et al., 2022; Orellana et al., 2023).

Eggshell translucency is primarily caused by the uneven deposition of protoporphyrins and other pigments, and is closely associated with maternal physiological status and genetic factors (Cheng et al., 2023). Accumulating evidence suggests that eggshell translucency is associated with lower hatchability compared with that of normal eggs (Cheng et al., 2023). Further molecular investigations have correlated translucency with the secretory regulation of oviduct epithelial cells, which may indirectly affect embryonic development by influencing eggshell formation, thereby reducing hatchability (Sun et al., 2026). However, some studies have demonstrated that although translucency affects hatchability, the effect is not statistically significant(Liu et al., 2021), which may be attributed to inter-breed variations. Moreover, translucency coexisting with insufficient eggshell mineralization, structural heterogeneity, or abnormal pore characteristics may indirectly reduce embryo viability by affecting gas exchange, water loss, and calcium supply to the embryo. This adverse effect may be more pronounced under prolonged storage or suboptimal incubation conditions (Orellana et al., 2023).

### Eggshell ultrastructure

**Fig. 2:**
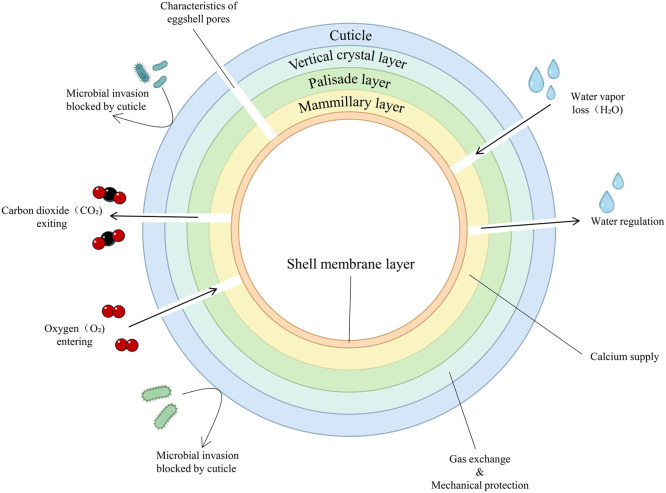
Schematic representation of eggshell ultrastructure and its functional roles in embryonic development. The eggshell consists of multiple layers, including the cuticle, vertical crystal layer, palisade layer, mammillary layer, and shell membrane. These layers collectively regulate mechanical protection, gas exchange, water loss, microbial barrier function, and calcium supply. The presence of pores across the eggshell enables oxygen diffusion and carbon dioxide release, which are essential for maintaining embryonic metabolism and development, ultimately influencing hatchability.

The eggshell is composed of a shell membrane layer, mammillary layer, palisade layer, vertical crystal layer, and a cuticle (Gautron et al., 2021), and its ultrastructural characteristics determine its mechanical strength and permeability of the shell (Athanasiadou et al., 2018; Biesek, 2023). As illustrated in Fig. 2, this multilayered architecture underpins key functional properties of the eggshell, including mechanical protection, gas exchange, and regulation of water loss. However, relying solely on macroscopic indicators, such as eggshell thickness or shell strength, is insufficient to comprehensively reflect eggshell function. The number, size, and uniform distribution of eggshell pores have a direct physiological role in regulating oxygen diffusion, carbon dioxide release, and moisture evaporation (Damaziak et al., 2023). Uneven pore distribution or a localized reduction in shell density may lead to localized hypoxia or embryonic dehydration, thereby affecting hatching synchrony and overall hatchability (Ipek and Sozcu, 2017). The mammillary layer is the primary source of embryonic calcium nutrition, supplying approximately 80% of the calcium required before hatching; consequently, its thickness is positively associated with hatchability, as a thicker mammillary layer provides sufficient calcium reserves, whereas thinning of this layer may directly impair embryonic development (Liao et al., 2013). The thickness of the vertical crystal layer is also positively correlated with the embryo survival rate, and its structural integrity may contribute to regulating the mechanical properties of the eggshell and gas exchange efficiency (Lunam and Ruiz, 2000).

In addition, eggshell pore characteristics are crucial for hatching synchrony. The spatial distribution, diameter, morphology, and their association with the eggshell crystal structure may significantly influence gas exchange efficiency (Damaziak et al., 2023). Excessively large size or uneven distribution of pores may locally impair gas exchange, resulting in early embryonic mortality or developmental retardation. Conversely, pores that are too small or exhibiting a pore density may restrict oxygen supply and increase embryonic metabolic stress.

### Cuticle

The cuticle is a transparent organic protective layer covering the outermost surface of the eggshell. As shown in Fig. 2, it serves as the first natural barrier between the hatching egg and the external environment, and a key structure that ensures embryonic development and hatchability (Chen et al., 2020). The cuticle regulates pore permeability to balance gas exchange and moisture loss. An intact cuticle prevents excessive or insufficient gas exchange under high altitude and low humidity conditions, providing a stable environment for CO₂ accumulation during early embryogenesis and O₂ supply during the mid-to-late stages. Cuticle damage may exacerbate the gas exchange imbalance-mediated adverse effects on hatchability (Juárez-Estrada et al., 2024; Okur et al., 2022). The gas exchange rate regulates the dynamic balance between O₂ uptake and CO₂ release. It directly influences embryonic development and survival probability (Agbehadzi et al., 2024; Fares et al., 2023; Juárez-Estrada et al., 2024; Okur et al., 2022). The cuticle optimizes gas exchange and hatchability by driving the pore permeability, whereas a gas exchange imbalance during incubation can reduce hatchability, particularly under high-altitude hypoxic conditions (Fares et al., 2023; Okur et al., 2022). Moreover, the cuticle prevents excessive evaporation of internal egg moisture through the shell pores, thereby maintaining optimal moisture loss during incubation (Huang et al., 2016) and preventing embryonic dehydration induced by excessive water loss or restricted gas exchange due to insufficient evaporation. Consequently, hatchability improves (Tawfeeq and Al-Samrai, 2021). However, damage to, or functional deterioration of, the cuticle may disrupt moisture regulation, reducing the hatchability (Nhan, 2025). Conversely, the synergistic interaction between cuticle and eggshell pore density ensures an appropriate range of moisture loss, and provides a stable internal environment for different stages of embryonic development, improving hatchability (Gong et al., 2024; Huang et al., 2016). Furthermore, the presence of the cuticle effectively inhibits the adhesion and penetration of surface contaminants on eggshells, thereby mitigating the negative effects of exogenous contaminants on hatchability (Vekic et al., 2021). Eggshell surface contamination provides a favorable environment for the proliferation of microorganisms, such as Escherichi*a coli* and *Staphylococcus*. These pathogens can penetrate the egg interior through shell pores and interfere with embryonic development, causing embryonic mortality and reducing hatchability (Mawaddah et al., 2025; Subagja et al., 2025).

## Effects of internal quality on hatchability

In contrast to the external egg quality, which primarily determines the integrity of the physical barrier, internal egg quality more directly drives the nutrient supply, safety, and biochemical environmental stability of the embryo throughout the incubation period; its role spans all stages of embryonic development. As shown in Fig. 1, internal egg quality affects hatchability mainly through regulating nutrient availability, protein composition, and microbial balance, thereby influencing the metabolic activity and developmental stability of the embryo.

### Characteristics of internal egg composition

The internal composition of the hatching egg is a critical determinant of hatchability; albumen and yolk serve as the two primary nutritional and functional components, with their chemical composition, proportion, and physicochemical properties directly influencing incubation outcomes by modulating the nutritional and physiological environment for embryonic development. Elevated levels of specific amino acids, such as lysine, threonine, and tryptophan, in the albumen are associated with significantly improved hatchability of eggs (Samiullah et al., 2015). Furthermore, albumen amino acids function not only as structural substrates for embryonic tissue formation but also participate in protein synthesis, energy metabolism, immune system development, and organogenesis throughout incubation. During embryogenesis, albumen proteins are progressively transferred into the amniotic fluid and utilized by the developing embryo, making the amino acid profile of albumen a key determinant of embryonic growth and viability(Gyawali and Goto, 2025; Biesek, 2023)Albumen is also rich in vitamins; however, its levels must be maintained within an optimal range, because both excessive and deficient concentrations can impair egg quality and consequently affect hatchability (Xiao et al., 2020). Vitamins such as vitamins A, D, E, and B-complex vitamins play essential roles in antioxidant defense, cellular differentiation, skeletal formation, and normal organ development of the embryo. Imbalances in vitamin concentrations may increase oxidative damage, impair embryonic development, and ultimately reduce hatchability (Tona et al., 2022; Oliveira et al., 2023) The Haugh unit (HU) is conventionally used as an indicator to assess albumen freshness and structural integrity. It influences hatchability by regulating mineral content in the albumen; a range of 81–91 is generally considered optimal for improved hatching performance (Wang, 2024). However, this range has been reported primarily in chicken breeder eggs and may not be universally applicable across different poultry species, breeder ages, or production systems. Previous studies have shown that the relationship between HU and hatchability can be influenced by factors such as flock age, egg storage duration, storage conditions, and incubation management practices, suggesting that species-specific optimal HU thresholds require further validation (Özlü et al., 2021; Tainika et al., 2023). Greater albumen height has a significantly positive effect on hatchability (Zhao, 2023); however, abnormally increased albumen height may suppress moisture loss during storage, thereby limiting the embryonic nutrient utilization and decreasing hatchability (Kucharska-Gaca et al., 2022).

Yolk is the primary source of energy, lipids, and fat-soluble vitamins for the embryo, and a moderately high yolk proportion promotes rapid growth and organ development during the mid to late stages of embryonic development, thus improving hatchability and chick quality (Milisits et al., 2010). However, an excessively high yolk proportion may increase the oxygen diffusion distance, particularly under conditions of low eggshell permeability, thereby increasing the risk of hypoxia and mortality during the late stages of embryogenesis. The relationship between yolk proportion and hatchability is regulated by multiple factors, and prolonged egg storage can significantly increase yolk proportion, adversely affecting embryo survival and reducing hatchability (Nhan, 2025).

The interior of an egg is not a sterile environment and comprises a microbial community that plays a crucial role in hatchability. Accumulating evidence suggests that low-abundance microbial communities can be detected in the albumen and yolk, and that their composition is associated with hatchability (Jin et al., 2022). Compromised eggshell barrier or inadequate disinfection can facilitate the penetration and rapid proliferation of pathogenic microorganisms within the egg, leading to embryonic infection and reduced hatchability. Previously, trimethylamine (TMA), an endogenous antimicrobial agent, has been reported to influence the internal antimicrobial system of eggs; it indirectly contributes to improved hatchability by inhibiting microbial infection during embryonic development (Shi et al., 2025).

### Abnormal internal components

Under normal conditions, the yolk and albumen function synergistically to support embryonic development, whereas abnormalities in egg composition disrupt this delicate balance, thereby significantly increasing the risk of hatching failure. Double-yolk eggs are the most common egg content abnormality, and their hatchability is significantly lower than that of single-yolk eggs. Studies have demonstrated that double-yolk eggs, even when containing a single embryo, face a higher risk of embryonic mortality owing to restricted developmental space, reduced gas exchange efficiency, and accumulation of metabolic waste (Banaszewska et al., 2023). Moreover, conditions such as decreased vitelline membrane strength, yolk rupture, and abnormally thin albumin can markedly affect the physical and biochemical microenvironments of the embryo, leading to hatching failure.

### Key protein composition and its genetic basis

In addition to the visible physical structures, genetic factors crucially influence the hatchability of eggs. Ovalbumin, a major storage protein, exhibits genetic polymorphisms that are significantly associated with hatchability and embryonic viability (Knaga et al., 2025). Different genotypes may indirectly regulate embryonic development by affecting the protein structural stability, nutrient release rate, and antioxidant capacity.

## Combined effects of egg quality and other factors

Egg quality is not a static trait; its dynamic changes from oviposition to pre-incubation are influenced by multiple regulatory factors that collectively determine the final hatchability. As highlighted in Fig. 1, these processes are further modulated by environmental and management factors, including storage duration, storage temperature, and incubation conditions, which interact with both external and internal egg quality traits. Storage duration, storage temperature, and early incubation handling practices are critical external regulatory factors that determine whether this intrinsic potential can be fully realized.

Storage duration is one of the most critical hatchability-related regulatory factors. It is widely recognized that hatched eggs maintain relatively high embryonic viability during short-term storage (5–7 d), whereas prolonged storage significantly reduces hatchability and chick quality (Abanikannda et al., 2025; Bouba et al., 2021; Gu et al., 2025; Nasri et al., 2020; Nhan, 2025; Nowaczewski et al., 2022; Reijrink et al., 2010), which is attributed to continuous moisture evaporation and gas exchange during storage, resulting in an increased albumen pH, loosening of the albumen structure, and a reduction in the Haugh unit, which disrupt the optimal microenvironment required for embryonic development (Biesek, 2023; Biesek et al., 2023; Dang et al., 2023). During storage, water is continuously lost through eggshell pores. Moderate water loss facilitates air cell formation and supports respiratory requirements in later embryonic stages, whereas excessive dehydration increases osmotic pressure and impairs embryonic cell activity and nutrient transport efficiency. Meanwhile, the loss of carbon dioxide leads to a gradual increase in albumen pH. A high pH environment can disrupt the gel structure of albumen, compromise the functional stability of key proteins, and potentially interfere with early embryonic cell division and tissue differentiation (Sherief et al., 2025). Importantly, the negative effects of prolonged storage appear to depend partly on the initial egg quality. It has been reported that deterioration of albumen quality during extended storage in Pekin duck eggs was accompanied by reduced hatchability and poorer duckling quality, suggesting that eggs with superior internal quality may better withstand storage-induced damage (Onbaşılar et al., 2007).

Low-temperature storage reduces the rates of metabolic activity and physicochemical reactions within eggs. Accumulating evidence suggests that storage at 7–13 °C can inhibit premature embryonic development while maintaining a relatively stable internal egg environment (Sherief et al., 2025; Wlaźlak et al., 2024). However, excessively low temperatures may induce chilling injury in the embryo, particularly when eggs are incubated immediately after storage without prewarming treatment. Meanwhile, rapid cooling after oviposition influences hatchability by delaying embryonic development; however, this effect varies with breeder hen age: this effect is detrimental in young flocks, reducing hatchability, whereas it is beneficial in older flocks, improving hatchability (Özlü and Elibol, 2023). This age-dependent response may be related to differences in egg composition and embryonic developmental status between younger and older breeder flocks. Eggs from young breeders generally contain smaller yolks and fewer nutrient reserves, making embryos more susceptible to developmental interruption caused by rapid cooling (Yadgary et al., 2010). In contrast, embryos from older breeders often exhibit more advanced development at oviposition and greater metabolic activity (Özlü et al., 2021). Therefore, rapid cooling may help synchronize embryonic development, reduce developmental variability among embryos, and decrease the incidence of developmental abnormalities, ultimately improving hatchability (Guinebretière et al., 2022). Furthermore, age-related alterations in eggshell characteristics and nutrient composition may contribute to differences in embryonic development and hatchability among breeder flocks of different ages (Avcılar et al., 2024).

The incubation temperature is critical for embryonic development. Excessively high incubation temperature enhances early embryonic mortality and reduces overall hatchability, whereas standard incubation conditions or short-term early high-temperature treatment can maintain relatively high hatchability (Avşar et al., 2022). However, embryonic responses to thermal conditions may also depend on egg quality, as variations in nutrient reserves and physiological status established before incubation can influence the capacity of embryos to tolerate temperature-related stress (Onbaşılar et al., 2014).

Moreover, differences in egg characteristics among avian species and genetic lines indicate that the relationships between egg quality, management practices, and hatchability are not universal, highlighting the need for species-specific incubation strategies (Onbaşılar et al., 2017).

These findings indicate that throughout the process, from oviposition to chick hatching, management factors such as time, temperature, and cooling rate collectively determine the final hatching outcomes through their interactions with egg quality.

## Regulatory strategies for improving egg quality and hatchability

Egg quality can be improved at different stages through nutritional regulation, including supplementation with organic trace elements, vitamin D₃, and antioxidants. It can also be enhanced through effective husbandry management, such as alleviating heat stress and ensuring breeder health, as well as optimization of hatching egg handling and incubation processes, including disinfection with plant essential oils, appropriate temperature control, and thermal conditioning during embryogenesis. As summarized in Fig. 1, these regulatory strategies act by improving both external and internal egg quality and optimizing their interaction with environmental conditions. Together, these interventions create more favorable conditions for embryonic development and significantly enhance hatchability.

### Management regulation of breeder flocks

Optimal feeding and effective management practices are essential external determinants ensuring that breeder flocks consistently produce high-quality eggs. Heat stress is the primary environmental stressor affecting poultry production efficiency and egg quality (Apalowo et al., 2024). High ambient temperatures can reduce feed intake and disrupt metabolic homeostasis, leading to decreased egg weight, thinner eggshells, and an increased incidence of abnormal eggs, which significantly reduce hatchability (Mack et al., 2013). Effective environmental management strategies, including evaporative cooling pad–fan systems, adequate water supply, maintenance of optimal lighting conditions, stable stocking density, and a low-disturbance environment, can effectively alleviate heat stress and associated behaviors, such as flock panic, thereby preventing the declines in eggshell strength and deterioration of internal egg quality and mitigating adverse effects on hatchability.

Inclusion of organic trace minerals, such as zinc, manganese, and copper, and antioxidants, such as vitamin E, vitamin C, and selenium, in the diet, is an effective strategy to improve eggshell quality and enhance hatchability (Dunn, 2023; Świątkiewicz et al., 2014). Organic trace minerals can activate key enzymes such as carbonic anhydrase, thereby promoting eggshell matrix formation and improving the microstructure, which enhances eggshell thickness and strength. Concomitant supplementation with antioxidants effectively protects embryonic cells from oxidative damage before the full establishment of the embryonic antioxidant system, reducing early embryonic mortality and supporting healthy development. Additionally, TMA precursors such as choline can be included in the diet as a regulatory measure to increase TMA deposition in the egg yolk through endogenous metabolic pathways, thereby indirectly promoting hatchability (Shi et al., 2025).

### Hatching egg handling and storage

Post-oviposition handling of hatching eggs and incubation conditions are the key regulatory factors of egg quality and embryonic development. Spraying 3% tea tree oil or lavender oil effectively disinfects the eggshell and improves the internal egg microenvironment by inhibiting weight loss during incubation and promoting embryonic development, thereby significantly enhancing hatchability and chick quality; these oils outperform formaldehyde and are considered a safe and effective alternative to formaldehyde fumigation. Essential oils reduce microbial invasion and water loss through eggshell pores, promoting favorable developmental conditions for embryos, which significantly reduce mortality and substantially improve hatchability (Iraqi et al., 2025). Disinfection of hatching eggs using 0.5% oregano oil and cumin oil effectively reduces the microbial consumption of egg contents, thereby minimizing egg weight loss during incubation and significantly enhancing both fertilized egg hatchability and set egg hatchability without adverse effects on embryonic development (Bekhet and Khalifa, 2022). Soaking duck eggs in cherry leaf extract that comprises antibacterial compounds such as flavonoids, saponins, and tannins can effectively reduce the bacterial load on the eggshell surface, reduce embryonic mortality, and improve hatchability (Ayuningtyas et al., 2022). Wet cleaning with a commercial food-grade detergent (P2) is highly effective in removing eggshell pathogens and enhancing hatchability (Mawaddah et al., 2025). The use of EPE packaging for short-term storage of hatching eggs can improve hatchability by maintaining albumen height and stabilizing microbial communities (Wang et al., 2026).

Although thermal stimulation does not directly improve the physical or biochemical characteristics of hatching eggs, it has been widely applied as an incubation management intervention to optimize embryonic development. Moderate thermal stimulation improves embryonic development and chick quality but does not significantly affect the final hatchability of fertilized eggs. However, thermal manipulation (TM) significantly increases hatchability of hatching eggs (TM group 94.50% vs. control 91.0%), shortens the incubation duration by approximately 6 h, and exhibits no significant adverse effects on chick hatching body weight. Specifically, TM treatment at 38.5°C and 55% relative humidity for 12 h daily during embryonic days (ED) 12–18 improved incubation performance compared with the control incubation conditions of 37.5°C and 55% relative humidity maintained throughout ED 12–21 . (Amaz et al., 2024). These results indicate that TM is an effective strategy for optimizing the incubation efficiency Such effects are mainly attributed to optimized incubation conditions and enhanced embryonic physiological responses, rather than direct improvements in egg quality.

Recent advances have further highlighted the importance of species-specific incubation management, particularly in goose production. Compared with chicken and duck eggs, goose eggs are characterized by larger egg size, thicker eggshells, lower shell conductance, and longer incubation periods, resulting in relatively lower hatchability and greater sensitivity to incubation conditions (Salamon, 2020). Recent studies demonstrated that scrubbing combined with 3% hydrogen peroxide spray effectively reduced eggshell microbial contamination and embryonic mortality while improving hatchability in goose (Wang et al., 2025). In addition, increasing egg-turning angles beyond the conventional 45° enhanced yolk utilization, promoted embryonic development, and improved hatchability and gosling quality (Guo et al., 2021). Cooling and spraying treatments during late incubation, which mimic the natural nesting behavior of geese, facilitate eggshell heat dissipation and regulate embryonic metabolism and water loss, thereby contributing to improved incubation outcomes (Salamon, 2020).

## Conclusion and perspectives

Overall, research indicates that egg quality regulates avian embryonic survival and development through multiple pathways, and serves as a fundamental determinant of hatchability. A concise summary of the major egg quality traits and their biological roles in embryonic development and hatchability is presented in Table 1. Eggshells function as a physical barrier and gas exchange interface; their external quality, comprising mechanical properties, structural density, and optical characteristics, such as color and translucency, collectively influences water loss, oxygen diffusion, and microbial invasion risk, thereby significantly affecting early embryonic survival and overall hatchability. In terms of internal quality, the yolk-to-albumen ratio, chemical composition, and genetic characteristics of key egg proteins determine the nutrient availability and supply as well as stability of the biochemical environment of the embryo, whereas abnormal egg content, such as double-yolk eggs, affects hatching through spatial and physiological constraints. Moreover, the egg microbiome, a dynamic variable, is increasingly recognized for its role in embryonic health and hatching outcomes.

**Table 1:** Major egg quality traits affecting hatchability and their underlying biological mechanisms.

**Egg quality trait**	**Main biological function**	**Influence on embryonic development and hatchability**	**Potential negative effects when abnormal**
Egg weight	Reflects nutrient reserve available to the embryo	Influences chick body weight and hatchability	Extremely small or large eggs may reduce hatchability
Eggshell thickness	Maintains shell strength and regulates gas exchange	Supports proper oxygen exchange and water balance during incubation	Excessively thin shells increase breakage, dehydration, and microbial invasion
Eggshell strength	Protects embryo from physical damage and environmental stress	Maintains incubation stability and embryo protection	Weak shells increase cracking and embryonic mortality
Eggshell color	Reflects shell mineralization and pigment deposition	May reflect shell mineralization status and incubation performance	Poor shell pigmentation may indicate shell quality defects
Eggshell translucency	Reflects eggshell ultrastructure and shell integrity	Associated with eggshell ultrastructural integrity and microbial barrier function	Increased translucency may increase bacterial penetration and reduce hatchability
Eggshell pore structure	Controls gas diffusion and water loss	Ensures appropriate oxygen supply and CO₂ exchange	Abnormal pore density may impair respiration and water regulation
Eggshell cuticle	Provides antimicrobial protection	Prevents microbial contamination during storage and incubation	Damaged cuticle increases bacterial invasion risk
Albumen quality (Haugh unit)	Maintains internal stability and nutrient protection	Supports embryo development and reduces microbial contamination	Albumen deterioration during storage reduces hatchability
Albumen physicochemical properties	Regulate viscosity, pH, and antimicrobial activity	Maintain stable embryonic environment	Excessive pH changes may impair embryonic development
Yolk-to-albumen ratio	Determines nutrient availability for embryo growth	Influences embryonic energy supply and chick quality	Imbalanced nutrient composition may impair embryo growth
Microbial contamination	Influences eggshell sanitation and embryo health	Low microbial load supports normal embryonic development	Severe contamination increases embryo mortality
Storage duration	Influences moisture loss and albumen deterioration	Appropriate storage duration maintains embryo viability	Long storage decreases hatchability and chick quality
Storage temperature	Regulates embryonic metabolic activity during storage	Proper temperature maintains embryo viability	Improper temperature accelerates quality deterioration
Incubation conditions	Affect embryonic metabolism and water balance	Proper incubation improves hatchability and chick quality	Improper temperature or humidity may impair embryonic development and hatchability

Egg quality is a dynamic trait influenced by multiple factors. Prolonged storage and inappropriate storage temperatures accelerate the deterioration of egg quality, resulting in moisture loss, increased pH, and reduced embryonic viability. Low-temperature storage and optimal incubation temperature can mitigate these adverse effects to some extent.

Future research should move beyond descriptive evaluations of egg quality traits and focus on elucidating the underlying mechanisms linking egg characteristics with embryonic development and hatchability. Given the complex interactions among breeder age, genetic background, egg storage conditions, and incubation management, integrated multifactorial studies are needed to identify the optimal conditions for maximizing hatchability. In addition, standardized methodologies and unified reporting criteria for assessing egg quality and hatchability should be established to improve comparability among studies and facilitate evidence synthesis. Moreover, the application of emerging technologies, including omics approaches, non-invasive imaging techniques, and artificial intelligence-based predictive models, may facilitate the identification of reliable biomarkers and improve hatchability prediction accuracy. In addition to the established determinants of hatchability, emerging factors such as the egg microbiome and endogenous antimicrobial metabolites (e.g., trimethylamine, TMA) may represent promising areas for future research. However, existing evidence remains preliminary, and additional studies are needed to elucidate their mechanistic roles and practical significance in hatchability regulation. Greater emphasis should also be placed on translating scientific findings into practical hatchery management strategies under commercial production conditions, thereby enhancing reproductive efficiency and sustainability in the poultry industry.

## CRediT authorship contribution statement

Yihan Wang: Conceptualization, Writing—original draft, Visualization, Literature collection and synthesis, Data curation. Zeyao Zhang: Writing—review & editing, Literature collection, Validation, Content refinement. Xuefeng Shi: Conceptualization, Supervision, Writing—review & editing, Funding acquisition, Project administration.

## Disclosures

The authors declare that they have no known competing financial interests or personal relationships that could have appeared to influence the work reported in this manuscript.
